# American, British, and Italian Online Information on the Health Risks Associated With Eating Meat: Cross-Sectional Study

**DOI:** 10.2196/81967

**Published:** 2026-06-23

**Authors:** Gianfranco Di Gennaro, Francesca Licata, Paolo Pittore, Aida Bianco

**Affiliations:** 1 Department of Health Sciences, University of Catanzaro “Magna Græcia”, Viale Europa, Catanzaro, 88100, Italy, 39 961 3697289; 2 Department of Medical and Surgical Sciences, University of Catanzaro “Magna Græcia”, Catanzaro, Italy

**Keywords:** cancer prevention, comparative analyses, diet sustainability, health risks, meat consumption, online information, Italy, United Kingdom, United States

## Abstract

**Background:**

The quality of online information regarding the risks associated with meat consumption could play a crucial role in shaping consumers’ behavior.

**Objective:**

This study aimed to investigate the quality of Italian, British, and American websites addressing this topic.

**Methods:**

A cross-sectional assessment of the top 100 British, Italian, and American web pages on the risks attributable to meat consumption was performed using the JAMA benchmarks tool, evaluating authorship by certified professionals and the inclusion of information on recommended meat consumption, potential meat substitutes, and coverage of issues such as diet sustainability and cancer, cardiovascular, and chronic disease prevention. Websites were then classified according to their stance toward meat consumption (neutral, promoting, or demonizing).

**Results:**

American and British websites were classified as high quality in 61% (61/100) and 78.1% (75/96) of cases, respectively, while only 22.3% (21/94) of Italian websites were classified as high quality. Multinomial regression showed that web pages with a demonizing stance toward meat consumption and those authored by certified health professionals were less likely to be Italian than American. Similarly, web pages discussing environmental risks and chronic diseases associated with excessive meat consumption were less likely to be Italian. Compared with American web pages, those promoting meat consumption and those authored by qualified professionals were less likely to be British. Web pages discussing chronic disease risks were also less likely to be British, whereas those mentioning cancer risks were more likely to be British.

**Conclusions:**

The widespread prevalence of poor online information quality, especially in certain countries, demands action. Promoting user education in assessing the reliability of websites and involving health professionals in this educational effort may represent viable strategies.

## Introduction

The link between meat consumption and health has been a significant area of concern in public health for many decades, and it gained even greater attention after the World Health Organization issued a warning in 2015 regarding the potential carcinogenicity of consuming red and processed meat [1-3]. Eating meat has been linked not just to cancer but also to cardiovascular diseases and other health issues such as pneumonia, diverticular disease, diabetes, and colon polyps [3-8].

At the turn of the 21st century, the 3 most trusted sources of health information were physicians, universities, and government agencies [9,10]. Today, however, consumers are increasingly turning to the internet and social media for nutrition-related information [11-16]. Among the public, young individuals, especially women, those with higher levels of education, and those living with chronic diseases, are more inclined to seek health information online, while older adults still prefer face-to-face consultations with health care professionals [9,17-19]. The quality of online health information is often suboptimal or poor, and the presence of web content created by unskilled or even malicious individuals remains a pressing issue [20-22]. In this regard, nutrition professionals and various public health organizations have expressed concerns about the potential harm caused by nutrition-related misinformation and its impact as a barrier to healthy eating behaviors [23].

Extensive literature has explored various methods for assessing the quality of information presented on websites, and several rating tools have been developed [24]. One of the most widely used methods is the JAMA benchmarks tool, a 4-point rating scale that assesses whether web content meets the following key criteria: “authorship” (ie, authors and contributors, affiliations, and relevant credentials are provided), “attribution” (ie, references and sources for all content are clearly provided, along with relevant copyright information), “disclosure” (ie, website ownership is prominently and fully disclosed, together with any sponsorship, advertising, underwriting, commercial funding arrangements or support, or potential conflicts of interest), and “currency” (ie, the dates on which content was posted and updated are indicated) [25].

Therefore, the aim of the study was to investigate and compare the quality of online information regarding health risks associated with meat consumption by examining the websites suggested by Google (Google LLC) in Italian, British, and American web searches.

## Methods

### Overview

In this cross-sectional study, the query “Is eating meat bad for you?” was entered into Google on December 14, 2022, and the first 100 organic search results from both American and British search engine results pages (SERPs) were retrieved using the web tool thruuu.com (A Thruuu Company) [26]. The process was repeated for the Italian web using the translation “Mangiare carne fa male?” A sample size of 100 organic search results per country was based on previous studies on online information quality [27-29]. This threshold aimed to ensure a balance between data completeness and the manageable workload of a manual website evaluation. Queries were selected through a preliminary elicitation phase involving a convenience sample of 10 adult native speakers from each country (Italy, United Kingdom, and United States). Participants were asked: “What would you write in the Google search bar to find information about the relationship between health and meat consumption?” (with the appropriate translation used for Italian respondents). Each participant could suggest up to 3 search terms. The most frequently cited queries were then identified for each country. In both Italy and the United Kingdom, the most common query was “Is meat bad for you?” while American participants cited “Is meat bad?” and “Is meat bad for you?” with equal frequency. As “Is meat bad?” and “Is meat bad for you?” yielded nearly identical search results on Google, we opted for the latter to ensure consistency across the 2 English-speaking countries. To validate the relevance and representativeness of the selected queries, we then asked all participants whether they considered the final choices appropriate and consistent with the aim of the study, even if their personal suggestions differed. Queries consisting of a more technical-scientific language were avoided because they are not representative of the online behavior of the average internet user and because they would lead to an overestimation of the quality of the information, as suggested in the literature [30,31].

To prevent the “filter bubble effect” (ie, the phenomenon that skews or limits the information an individual user encounters online), the Google searches were carried out in a “private mode” window through the Google Chrome browser after deleting the search history and cache and using a never-before-used ad hoc Google account. Results directing to pages under maintenance or to YouTube videos were removed.

The trustworthiness of each web page was measured using the JAMA benchmarks. A score was calculated by attributing 1 point to each of the 4 JAMA benchmarks criteria when satisfied. The web pages reporting a score of 3 or 4 were classified as informative, high-quality web pages, as suggested by the literature [32,33].

To further assess the completeness of the information provided, a panel of 5 nutritionists external to the study has identified the following 6 topics the websites should ideally inform users about: “guidelines” (ie, precise quantitative indications corresponding to the meat requirement), “environmental sustainability” (ie, the impact of meat production on the environment), “cancer prevention” (ie, the risk of cancer associated with high meat intake), “cardiovascular prevention” (ie, the risk of cardiovascular diseases associated with high meat intake), “chronic diseases prevention” (ie, the risk of chronic diseases associated with high meat intake), and “meat substitutes” (ie, clear indications regarding meat alternatives in the diet).

In addition, the authority of each website was assessed (ie, the content was created by a certified health professional). The websites were categorized into the following 6 categories (as reported in previous research): “journalistic,” “professional,” “governmental,” “nonprofit,” “commercial,” and “health portal” [34]. Finally, each website was rated for its stance on meat consumption. More precisely, a website was classified as “demonizing” if it explicitly warned against meat consumption, focused predominantly on its risks (such as cancer, cardiovascular disease, or environmental harm), or recommended reducing or eliminating meat from the diet. Conversely, a “promoting” stance was attributed to websites that emphasized the health benefits of meat, such as its role as a source of essential nutrients or proteins, while downplaying potential risks or explicitly encouraging its consumption. Finally, websites were considered “neutral” if they presented a balanced view, discussing both risks and benefits without expressing a clear evaluative stance, or if they avoided making explicit recommendations altogether.

**Figure 1.**
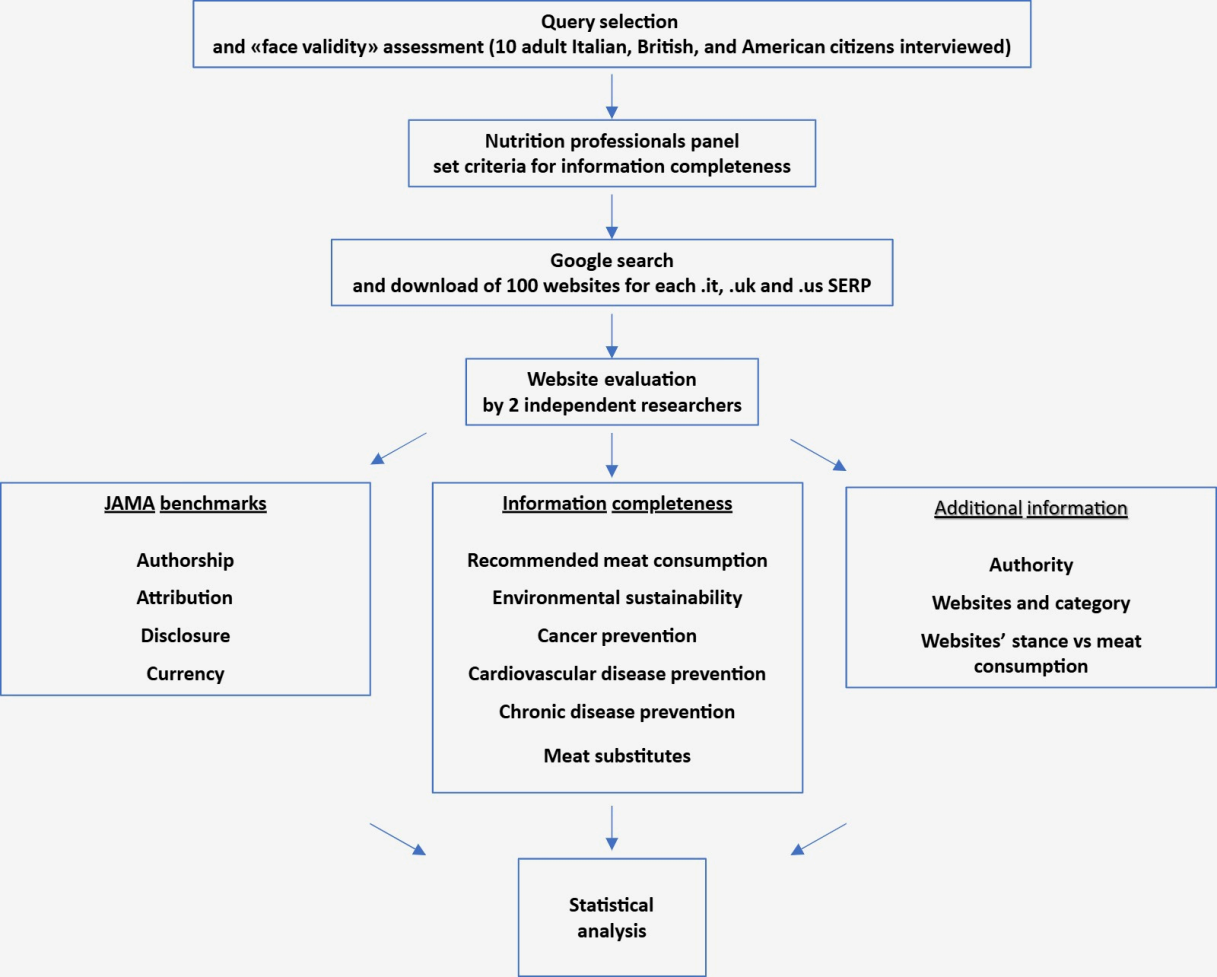
Overview of the study’s workflow. SERP: search engine results page.

Websites were independently analyzed by 3 researchers (GDG, PP, and FL). A fourth author (AB) arbitrated in case of disagreement. To minimize the risk of overevaluation, given that evaluators were aware of the study’s purpose, we applied the “3-click rule”: evaluators were instructed not to go beyond 3 clicks after landing on a website [35]. This constraint was designed to simulate realistic online behavior and to limit access to information that would not typically be reached by an average user. This approach has also been adopted in other similar studies [34]. Figure 1 provides an overview of the study’s workflow. This study analyzed only publicly available online content. No individual or sensitive data were collected or processed. Therefore, no ethics approval or informed consent was required.

The reporting of this study complies with the Strengthening the Reporting of Observational Studies in Epidemiology (STROBE) standards for reporting observational studies [36].

### Statistical Analysis

Data were summarized using counts and percentages, as all variables were categorical. The overall and country-specific proportions of high-quality websites were estimated with 95% CIs. A multinomial regression model was used to investigate the differences between American, British, and Italian websites. In this model, the “country” variable served as the dependent variable. The web pages’ quality classification (high or nonhigh quality), website category, stance on meat consumption, and all additional evaluation criteria (authority, guidelines, diet sustainability, cancer and cardiovascular prevention, and meat substitutes) were analyzed as independent variables.

Similarly, to define the quality characteristics of web pages classified as high quality, a binomial logistic regression model was used. In this model, website classification as high quality or not high quality served as the dependent variable. In addition to the predictors described above, website ranking in Google’s SERP was included as an independent variable. The adequacy of both statistical models was evaluated by calculating the area under the curve (AUC), visually inspecting the standardized Pearson residuals, and performing the Hosmer-Lemeshow goodness-of-fit test.

Furthermore, to mimic the behavior of average web users, we carried out a sensitivity analysis restricting the comparison to only the first 10 results of the 3 SERPs, as this is the number of top sites mostly consulted from SERP searches [37,38]. In this case, because a multivariable assessment was not feasible due to the low number of websites, between-country comparisons were performed for each collected variable using the Fisher exact test to take into account small (<5) size cells. No missing data handling strategy was applied, as variables were defined such that the absence of information on a given topic was coded as “not reported” rather than treated as missing. Statistical analyses were performed using the statistical package Stata (version 19; StataCorp LLC) [39]. Multinomial and logistic forest plots were produced in R software (version 4.0.5; R Foundation for Statistical Computing) [40].

### Ethical Considerations

This study involved the assessment of publicly available web pages and did not include the collection or processing of any personal or identifiable data. Therefore, ethics approval was not required, and the study was exempt from institutional review board oversight in accordance with applicable institutional guidelines.

## Results

### Sample Description

Out of the 300 downloaded web pages, 6 Italian and 4 British websites were removed because a UK site linked to a YouTube video, while the remaining 9 removed sites were under maintenance. Overall, “journalistic” (n=122, 42.1%) and “health portal” (n=99, 34.1%) websites were the most commonly displayed in the Google SERPs, followed by “professional” (n=38, 13.1%) and “commercial” (n=19, 6.6%) websites. As “government” and “nonprofit” websites were least frequently disclosed (n=12, 4.1%), they were combined into a single category labeled “other” (Table 1).

**Table 1.** Distribution of information quality, completeness of the information provided, and website characteristics, stratified by country.

	Italy (n=94), n (%)	United Kingdom (n=96), n (%)	United States (n=100), n (%)	Overall (N=290), n (%)
The website meets the following JAMA benchmarks				
Authorship	32 (34)	81 (84.4)	76 (76)	189 (65.2)
Attribution	20 (21.3)	51 (53.1)	50 (50)	121 (41.7)
Disclosure	29 (30.9)	68 (70.8)	57 (57)	154 (53.1)
Currency	74 (78.7)	91 (94.8)	91 (91)	256 (88.3)
High quality (JAMA score>2)	21 (22.3)	75 (78.1)	61 (61)	157 (54.1)
The author is a qualified health professional	34 (36.2)	59 (61.5)	75 (75)	168 (57.9)
The website reports				
Recommended meat consumption	35 (37.2)	62 (64.6)	52 (52)	149 (51.4)
Risks for environmental sustainability	15 (16)	21 (21.9)	36 (36)	72 (24.8)
Risk of chronic diseases	33 (35.1)	56 (58.3)	64 (64)	153 (52.8)
Risk of cardiovascular diseases	30 (31.9)	54 (56.3)	52 (52)	136 (46.9)
Risk of cancer	15 (16)	45 (46.9)	30 (30)	90 (31)
How to replace meat with other foods	20 (21.3)	47 (49)	41 (41)	108 (37.2)
Website category				
Commercial	13 (13.8)	2 (2.1)	4 (4)	19 (6.6)
Journalistic	36 (38.3)	47 (49)	39 (39)	122 (42.1)
Health portal	34 (36.2)	38 (39.6)	27 (27)	99 (34.1)
Professional	6 (6.4)	9 (9.4)	23 (23)	38 (13.1)
Other (government or nonprofit)	5 (5.32)	0 (0)	7 (7)	12 (4.1)
Stance on meat consumption				
Neutral	36 (38.3)	52 (54.2)	35 (35)	123 (42.4)
Promoting	32 (34)	14 (14.6)	21 (21)	67 (23.1)
Demonizing	26 (27.7)	30 (31.1)	44 (44)	100 (34.5)

Almost two-fifths of the Italian websites were categorized as “journalistic” (36/94, 38.3%), followed by “health portal” (34/94, 36.2%), “commercial” (13/94, 13.8%), and “professional” (6/94, 6.4%). In the British SERP, “journalistic” websites were predominant (49/96, 49%), followed by “health portal” (38/96, 39.6%) and “professional” (9/96, 9.4%). There were only a few “commercial” (2/96, 2.1%) websites. Similarly, “journalistic” websites were the most common (39/100, 39%) in the American SERP, followed by “health portal” (27/100, 27%), “professional” (23/100, 23%), and “commercial” (4/100, 4%) websites. In all 3 SERPs, websites with a “neutral” stance on meat consumption were consistently the most prevalent. In Italy, “neutral” websites accounted for 38.3% (36/94), followed by “promoting” at 34% (32/94) and “demonizing” at 27.7% (26/94). In the British SERP, the “neutral” stance was the most common, with 54.2% (52/96), followed by “promoting” at 14.6% (14/96) and “demonizing” at 31.1% (30/96). In the American SERP, “neutral” websites were also the most common (35/100, 35%), followed by “promoting” at 21% (21/100) and “demonizing” at 44% (44/100).

### JAMA Benchmarks Criteria and Overall Quality

More than half (157/290, 54.1%) of the websites assessed were deemed high quality. In particular, American and British websites demonstrated high quality in 61% (61/100; 95% CI 50.7%-70.6%) and 78.1% (75/96; 95% CI 68.5%-85.9%) of instances, respectively, whereas only 22.3% (91/94; 95% CI 14.4%-32.1%) of Italian websites met this standard. The “currency” criterion was most frequently met (256/290, 88.3%), followed by “authorship” (189/290, 65.2%), “disclosure” (154/290, 53.1%), and “attribution” (121/290, 41.7%). However, substantial variations in adherence to JAMA benchmarks were observed across the 3 SERPs. In the Italian SERP, 34% (32/94) of websites met the “authorship” criteria, whereas in the British and American counterparts, this percentage notably increased to 84.4% (81/96) and 76% (76/100), respectively. Regarding “attribution,” 21.3% (20/94) of Italian websites provided proper references, compared with 53.1% (51/96) of British websites and 50% (50/100) of American websites. The “disclosure” criterion was met by 30.9% (29/94) of Italian websites, while this percentage was notably higher among the British (68/96, 70.8%) and American (57/100, 57%) websites. For “currency,” 78.7% (74/94) of Italian websites were up to date, compared with 94.8% (91/96) of British and 91% (91/100) of American websites. High-quality websites accounted for 22.3% (21/94) in Italy, 78.1% (75/96) in the United Kingdom, and 61% (61/100) in the United States.

### Authority and Additional Criteria

Overall, only 57.9% (168/290) of the content on the analyzed websites was authored by certified professionals. In particular, only 36.2% (34/94) of Italian websites and 61.5% (59/96) of British websites met this criterion, while American websites met the “authority” criterion in 75% (75/100) of cases. Approximately half of the websites (149/290, 51.4%) provided the recommended meat intake. Among these, Italian websites represented the smallest proportion, with 37.2% (35/94) meeting the criterion, compared with 64.6% (62/96) of British websites and 52% (52/100) of American websites. Environmental sustainability risks were the least addressed topic overall (72/290, 24.8%). Italian websites covered this aspect in only 16% (15/94) of cases, compared with 21.6% (21/96) of British websites and 36% (36/100) of American websites. Regarding health risks, noncommunicable diseases (153/290, 52.8%), cardiovascular diseases (136/290, 46.9%), and cancer risk (90/290, 31%) were the most frequently cited. Italian websites addressed noncommunicable diseases in 35.1% (33/94) of cases, cardiovascular diseases in 31.9% (30/94), and cancer risk in 16% (15/94). British websites were more likely than American websites to emphasize cancer risk (45/96, 46.9% vs 30/100, 30%) and cardiovascular diseases (54/96, 56.3% vs 52/100, 52%). Conversely, American websites provided more information on noncommunicable diseases (64/100, 64% vs 56/96, 58.3%) compared to British websites. Alternatives to meat were indicated in 37.2% (108/290) of cases. Coverage of this topic was observed in 21.3% (20/94) of Italian websites, compared with 49% (47/96) of British websites and 41% (41/100) of American websites.

### Adjusted Between-Country Differences

#### Italian vs American Websites

**Figure 2.**
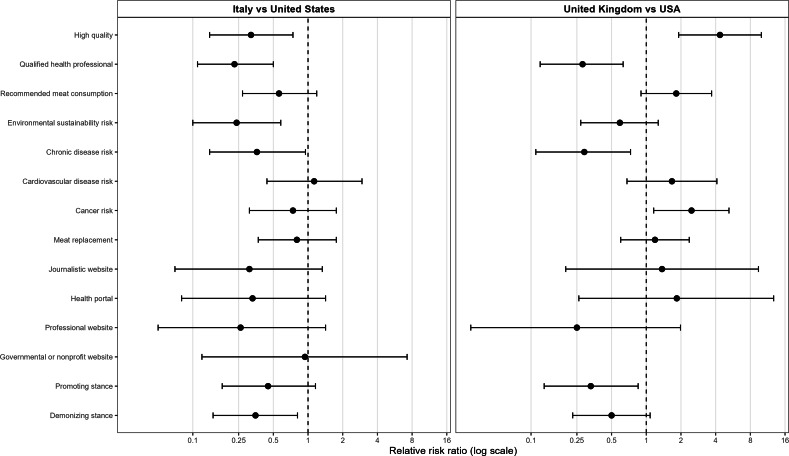
Multinomial regression model predicting differences between American, British, and Italian websites. The model demonstrated good discrimination, with area under the curve (AUC) values of 0.81 for the United Kingdom vs the United States and 0.84 for Italy vs the United States. Hosmer-Lemeshow goodness-of-fit tests indicated adequate model fit (United Kingdom vs United States: *P*=.15; Italy vs United States: *P*=.36).

As shown in the multinomial regression analysis (Figure 2), high-quality web pages were significantly less likely to be Italian rather than American (relative risk ratio [RRR] 0.32, 95% CI 0.14-0.74).

Similarly, web pages authored by qualified health professionals were less likely to be Italian rather than American (RRR 0.23, 95% CI 0.11-0.50). No significant differences emerged between Italian and American web pages with respect to reporting recommended meat consumption or risks related to environmental sustainability.

Web pages discussing the risk of chronic diseases were less likely to be Italian rather than American (RRR 0.36, 95% CI 0.14-0.95). No significant differences were observed between Italian and American web pages in the discussion of cardiovascular disease risk, cancer risk, or advice on meat replacement. Similarly, no statistically significant differences were found in the distribution of website categories between Italian and American SERPs.

Web pages adopting a demonizing stance toward meat consumption (vs a neutral stance) were significantly less likely to be Italian rather than American (RRR 0.35, 95% CI 0.15-0.81).

#### British vs American Websites

Web pages classified as high quality (vs lower quality) were significantly more likely to be British rather than American (RRR 4.36, 95% CI 1.91-9.97). However, web pages authored by qualified health professionals were less likely to be British rather than American (RRR 0.28, 95% CI 0.12-0.63). The reporting of recommended meat consumption, the risk for environmental sustainability, the risk for cardiovascular diseases, and the replacement of meat with other foods did not show significant differences between British and American websites. Web pages discussing the risk of chronic diseases were less likely to be British rather than American (RRR 0.29, 95% CI 0.11-0.73). In contrast, web pages discussing the risk of cancer were more likely to be British rather than American (RRR 2.47, 95% CI 1.16-5.22). Regarding website categories, no statistically significant differences emerged between British and American SERPs. Web pages promoting meat consumption (vs a neutral stance) were significantly less likely to be British rather than American (RRR 0.33, 95% CI 0.13-0.85).

The multinomial model exhibited satisfactory performance when comparing the American reference websites with both the British (AUC 0.81; goodness-of-fit *P*=.15) and Italian (AUC 0.84; goodness-of-fit *P*=.36) websites.

### Predictors of Websites’ Quality

**Figure 3.**
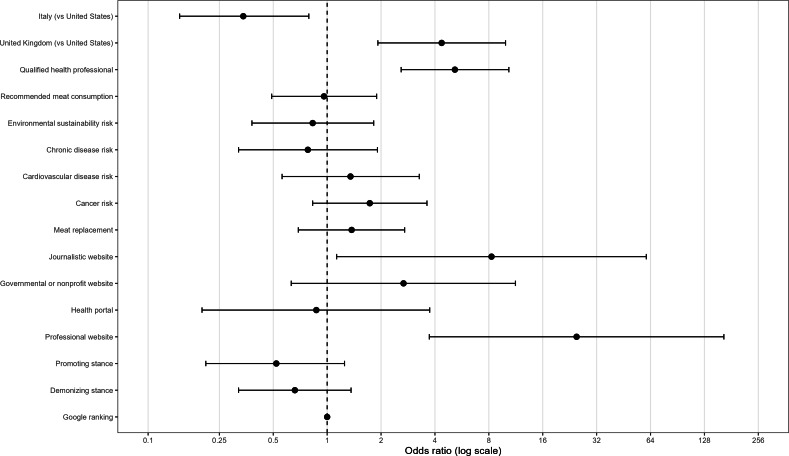
Binomial logistic regression model predicting classification of websites as “high quality.” The model demonstrated excellent discrimination (area under the curve=0.88), and the Hosmer-Lemeshow goodness-of-fit test indicated adequate model fit (*P*=.87).

When compared to their American counterparts, the logistic regression analysis (Figure 3) confirmed that Italian websites had a significantly lower likelihood of being classified as high quality (odds ratio [OR] 0.34, 95% CI 0.15-0.79), while British websites exhibited higher quality (OR 4.36, 95% CI 1.92-9.92).

As expected, web pages authored by qualified health professionals were associated with significantly higher quality (OR 5.17, 95% CI 2.59-10.35) compared to those authored by unqualified individuals. On the other hand, discussions related to meat consumption recommendations, environmental sustainability risks, chronic disease risks, cardiovascular disease risks, cancer risks, and meat replacement strategies were not linked to higher quality.

Websites categorized as “journalistic” (OR 8.28, 95% CI 1.13-60.62) and “professional” (OR 24.73, 95% CI 3.72-164.42) were significantly more likely to be classified as high quality compared to commercial websites.

Finally, the stance on meat consumption and Google ranking did not significantly impact the likelihood of being classified as high quality.

### Restricted Analysis of the Top 10 Indexed Web Pages

When considering only the top 10 Google-indexed websites, the distribution among the 3 SERPs, for most of the criteria analyzed in the study, appear to be similar to the results emerged from the full-dataset analysis. In general, Italian websites continued to exhibit lower compliance with high-quality, “authority,” and information completeness criteria (data not shown). More specifically, significant differences emerged in relation to the communication of chronic disease risks, with only 3 Italian websites addressing the topic, compared to 9 in both the British and American SERPs (*P*=.005). Similarly, information regarding meat replacement was found on 2 Italian websites, 8 British websites, and 6 American websites (*P*=.04). No significant differences emerged among stance on meat consumption or the distribution of the website category.

## Discussion

### Principal Findings

It is well known that the internet has given users convenient access to various types of online information resources, including health-related information [19,41,42]. Considering how easy it is to search on Google and how difficult it can be to consult health professionals, it is not surprising that control over health information related to individuals has greatly decreased. This has elicited uncertainty about the role of the internet as a tool to improve health care.

This study revealed significant deficiencies regarding essential information (eg, recommendations on the optimal amount of meat to consume and the likelihood of developing cancer) that websites should provide when discussing excessive meat consumption and associated risks. In this regard, it is crucial to highlight that warnings about health risks are crucial when the goal of communication is to reduce meat consumption, as demonstrated in a recent meta-analysis [43]. It is important to note that Italian websites exhibited a notable lag in the completeness of information when compared to their counterparts in the British and American SERPs. Furthermore, it is concerning that, in all 3 countries, much of this information was disseminated by individuals who lack certification as nutritionists and/or health experts.

The results presented in this study are consistent with findings from previous research in the literature. A study conducted by Hirasawa et al [44] demonstrated that websites providing information on a healthy diet only partially adhered to the guidelines. Similarly, a review by Denniss et al [45] in 2003 revealed that online information about nutrition was of poor quality in nearly 50% of the papers where accuracy and information quality were assessed. The discrepancy in the quality of online information is also detectable in the literature. In fact, a comprehensive evaluation of online health information revealed lower quality in the 2 larger middle-income countries, China and India, compared to the United States as a benchmark [46]. Similarly, another study indicated that English websites offered superior information in urological oncology when compared to their German, Spanish, and French counterparts [47]. Moreover, an international assessment of the quality and content of internet information on osteoarthritis showed that Western countries such as the United States, Mexico, France, and Germany offered a higher level of information than Eastern countries such as China, Japan, and Indonesia [48].

Identifying the causes of these divergences is a complex task. Numerous factors can play a role, including the sociocultural and health characteristics of the population across the different countries. For instance, in light of the findings of this study, a paradox may be considered: in the United Kingdom and the United States, where historically higher rates of obesity and overweight have been observed compared with Italy, there is a greater need for comprehensive and high-quality nutritional information for preventive purposes. Similarly, it is not surprising that a cuisine-focused country such as Italy yields a higher percentage (32/94, 34%) of websites advocating meat consumption compared to the United Kingdom and the United States (14/96, 14.6% and 21/100, 21%, respectively).

Finally, in our study, website quality is not related to site ranking in the Google SERP. In this regard, this result confirms what emerges from other studies in which the most reliable websites do not rank at the top of search engine results, including Google, Bing, and Yahoo [9].

Addressing the potential harm caused by the low quality of health information identified in this study requires a multifaceted approach. First, in accordance with suggestions from several authors, we believe it is essential to educate users on how to autonomously assess the quality of the information they access online. This can only be achieved by incorporating instruction on proper internet use into the early stages of formal education [9]. Second, this paradigm shift, wherein individuals engage in self-education within the realms of nutrition and health care, places a responsibility on health care professionals to assume the role of educating patients on how to accurately evaluate online information, as observed in a study evaluating the quality of online information regarding breast cancer treatment options [49]. Finally, it is crucial to have search engines that, in contrast to current practices, also prioritize the most reliable and authoritative websites, rather than solely those with the most relevant information, irrespective of their accuracy [50].

### Strengths and Limitations

To the best of our knowledge, there are no studies in the existing literature that specifically analyze the quality of information available on the internet regarding the health risks associated with meat consumption while comparing results across 3 different countries. Additionally, a potential strength of this study lies in its attempt to faithfully replicate online user behavior by selecting Google queries after surveying potential users and adhering to the “3-click rule.” Nonetheless, it is important to interpret the results of this study, considering its potential limitations. First, the assessment of website quality relied solely on JAMA benchmarks, one of the numerous tools aimed at evaluating the quality of online information. Second, we used a single Google query for website collection, and the additional analysis of similar queries might have yielded additional insights. However, we have verified that alternative queries such as “is eating meat harmful?” or “health risks of eating meat,” along with their corresponding Italian translations, lead to highly overlapping results with those obtained with the queries used in this study. Finally, although our assessment addressed quality and completeness, we did not specifically investigate the presence of potentially harmful content. This could represent an important aim for future investigations.

### Conclusions

The results of this study underscore the necessity, particularly in Italy compared to the United States and the United Kingdom, for enhanced national governmental oversight of the accuracy of online information dissemination, beyond just issues related to meat consumption risks. Additionally, the lack of search engines that prioritize content based on quality and comprehensiveness represents a significant limitation. An immediately actionable strategy involves engaging health care professionals in educating users on how to autonomously evaluate the quality of health care information.

## Abbreviations

AUC: area under the curve
OR: odds ratio
RRR: relative risk ratio
SERP: search engine results page
STROBE: Strengthening the Reporting of Observational Studies in Epidemiology
